# Management of HBV Infection During Immunosuppressive Treatment

**DOI:** 10.4084/MJHID.2009.025

**Published:** 2009-12-22

**Authors:** Alfredo Marzano

**Affiliations:** Division of Gastroenterology and Hepatology, San Giovanni Battista Hospital, Turin, Italy

## Abstract

The literature on hepatitis B virus (HBV) in immunocompromised patients is heterogeneous and refers mainly to the pre-antivirals era. Currently, a rational approach to the problem of hepatitis B in these patients provides for: a) the evaluation of HBV markers and of liver condition in all subjects starting immunosuppressive therapies (baseline), b) the treatment with antivirals (therapy) of active carriers, c) the pre-emptive use of antivirals (prophylaxis) in inactive carriers, especially if they are undergoing immunosuppressive therapies judged to be at high risk, d) the biochemical and HBsAg monitoring (or universal prophylaxis in case of high risk immunosuppression, as in onco-haematologic patients and bone marrow transplantation) in subjects with markers of previous contact with HBV (HBsAg-negative and antiHBc-positive), in order to prevent reverse seroconversion. Moreover in solid organ transplants it is suggested a strict adherence to the criteria of allocation based on the virological characteristics of both recipients and donors and the universal prophylaxis or therapy with nucleos(t)ides analogs

## Introduction:

Hepatitis B virus infection is a major public and medical concern. Two billion people are overt carriers of HBV worldwide; of them, 360 million suffer from chronic HBV infection and over 520,000 die each year, 50,000 from acute hepatitis B and 470,000 from cirrhosis or liver cancer. Moreover many subjects have only markers of previous contact with the HBV (antiHBc+/− antiHBs), which can indicate an Occult HBV Infection (OBI).

Immunodepression due to the underlying disease or to drugs used in immunosuppressive, anticancer therapy and in organ transplants can influence the hepatitis B virus (HBV), both in terms of reactivation and in terms of the acceleration of a pre-existing chronic hepatitis. In this situation the possibility of HBV relapse has been known for years, with clinical manifestations ranging from selflimiting anicteric to fulminant forms or to chronic hepatitis with an accelerated clinical course towards liver decompensation. Hepatitis reactivation may influence the continuation of the specific treatments and the survival of immuno-depressed or transplanted patients^1^.

The risk of clinical events is mainly observed in overt carriers of HBV, but can also develop in the OBI condition which has been widely described in the literature of the last decade.^2^

Progress in the diagnostic procedures of the various virological conditions associated with HBV, the recent availability of effective antiviral treatments, the growing incidence of immunocompromised patients attributable to the evolution of immunosuppressive therapies and organ transplants and the expectation of an important future increase of HBV reactivation have brought this problem to the fore, although the rational approach and management of these patients is still debated.

## Definitions

### Virological characteristics:

Persistent HBV infection is defined as overt when the hepatitis B surface antigen (HBsAg) is present in amounts well-detectable by sensitive immune assays and occult in HBsAg-negative subjects with evidence of intrahepatic and/or serum HBV DNA.^2^ In occult carriers, HBsAg can be completely absent (real OBI) or undetectable for very low amounts or polymorphisms (false OBI).

A. HBV carriers (HBsAg-positive). In accordance with the international definitions, they can be identified as: 1) active carriers, in presence of HBeAg or of anti-HBe antibodies and of a viral load ≥ 2–20,000 IU/ml; this condition is associated with the presence of hepatic disease in the most part of cases, or 2) inactive carriers, in case of subjects HBeAg-negative and antiHBe-positive, whose alanine aminotransferase (ALT) levels are persistently within the normal range, HBV DNA below 2,000 IU/ml in the most part of cases and IgM antiHBc levels < 0.20 IMx Index. In the majority of these subjects the histological finnding, when available, does not reveal a significant liver disease (necro-inflammatory activity < 4 HAI), while in a small minority of cases it is possible to observe the effects of a chronic liver disease which became silent spontaneously or following antiviral treatment^3^,^4^.

B. Occult HBV carriers (HBsAg-negative). The difficulty in determining HBV DNA in the liver biopsy (frequently not justified in subjects without clinical signs of hepatitis), the rare presence of detectable viremia in serum even with sensitive techniques, and the frequent presence in occult carriers of markers of previous contact with the HBV (antiHBc+/− antiHBs), leads one to consider all anti-HBc (anti-core)-positivesubjectsas potential occult carriers. Instead there are no serum determinants in the minority (about 20%) of occult carriers who are negative for all HBV markers.

### Virological events:

In HBV carriers (occult or overt) the following virological events are considered significant: 1) in anti-core subjects the reemergence of HBsAg(sero-reversion), 2) in inactive carriers the appearance of a significant viremia (≥20,000 IU/ml) (reactivation), as this is frequently associated with liver damage due to HBV, 3) in active carriers the persistence of a significant viremia (> 20,000 IU/ml in HBeAg positive patients and > 2,000 IU/ml in HBeAg negative subjects) (activity), as this is frequently associated with progression of liver damage due to HBV, 4) in all the virological categories (whether or not during prophylaxis or therapy with antivirals), the increase in at least one logarithm of HBV DNA, compared to its nadir, reconfermed in two consecutive serum tests during monitoring (virologic breakthrough) (Table 1)^4^.

### Clinical definitions:

The assessment of chronic liver disease is the fundamental event of the diagnostic picture (baseline) (Table 2) and requires the use of all the instruments usually utilised in hepatology including, if necessary, trans-cutaneous or trans-jugular liver biopsy in subjects with coagulation problems (for example patients with blood or kidney diseases).

The baseline diagnosis of the disease is pivotal in the choice of which treatment to adopt, as the risk of severe complications is related to the severity of the underlying liver disease^5^.

In order to standardize the deÞ nitions the following terms were suggested: 1) infection (not necessarily associated with reactivation of hepatitis) in the case of the detection of HBV DNA by sensitive HBV assays and/or of HBsAg in patients in whom these markers were originally negative, 2) reactivation of hepatitis B (hepatitis), in the presence of a significant viremia and ALT levels above the upper normal value.

## Treatment Strategies:

The term prophylaxis was used to mean treatment with antiviral drugs of an inactive or occult infection, with the aim of preventing hepatitis reactivation. Prophylaxis was defined as: 1) universal prophylaxis (UP), if it is carried out on the entire population potentially at risk (inactive carriers and/or anti-core), 2) or targeted prophylaxis (TP), if it is subordinate to the appearance of infection markers (HBV DNA and/or HBsAg) in the absence of hepatitis reactivation (Table 3). Therapy (T) was understood to mean the treatment of hepatitis B (i.e. chronic hepatitis in active carriers or hepatitis reactivation)

## Treatment Options:

In Italy the following drugs are available at present: interferons, either standard or peghilated (both little tolerated in the condition of immunodepression, especially in transplant patientsfor the potential risk of rejection) and the nucleos(t)ides analogs(NAs), which currently include lamivudine, adefovir-dipivoxil, entecavir, telbivudine and tenofovir and and emtricitabine for patients with HBV-HIV co-infection.

In naive patients lamivudine, which has a considerable antiviral effect, frequently (50–60% at 4 years, low genetic barrer) induces the selection of lamivudine-resistant mutants in locus YMDD of the polymerase gene (YMDD). However, adefovirdipivoxil has a low antiviral effect but induce a lower selection of mutants, while Telbivudine is more potent with an intermediate genetic barrer. Finally, third generation NAs (Entecavir and Tenofovir) have both a high potency and a high genetic barrer^3^.

Data from experience in liver transplanted and HIV patients have shown a relation between the original viremia, the degree of immunosuppression and the selection of mutants during prophylaxis with lamivudine.^9^,^10^ Consequently a careful monitoring of the response to treatment and of the resistance is suggested in immunocompromised patients treated with Nas.

Hereafter are reported the statements of the Italian guidelines referred to hepatitis B and recently updated with a special attention to the different therapeutic options available nowadays.^8^

Screening. It is recommended that all immunocompromised patients and those candidate to chemotherapy, immunosuppressive therapy and/or transplantion are screened for HBsAg and anti-HBc. Seronegative patients should be vaccinated preferibly with a reinforced course of vaccination for the diminished vaccinal response linked to the immunocompromission.

Chronic carriers with active HBV replication (HBV DNA > 2.000 IU/mL). They should be treated as immune-competent patients. NAs are the first choice, regardless of the clinical setting (oncology, haematology, rheumatology, nephrology, gastroenterology, dermatology, solid organ transplantation). Pegylated interferon is contraindicated in most cases. NAs with high potency and low resistance should be used, such as Entecavir or Tenofovir. Telbivudine could be considered in those with HBV DNA < 2,000,000 IU/ml.

Close virologic monitoring is mandatory during immunosuppression. The addition of a second drug (a nucleotide in patients treated with a nucleoside and vice versa) is advisable in cases of incomplete virologic response, or primary non-response to monotherapy. In immunocompromised patients the dose of NA(s) should be adjusted according to the renal function, co-morbidities and drugs interactions.

Inactive HBsAg carriers (HBV DNA persistently < 2,000 IU/ml). In patients undergoing solid organs transplant or autologous or allogenic bone marrow transplantation or high risk immune suppressive treatment (anti-TNF, anti-CD20, anti-CD56, medium/high dose of steroids (>10 mg/die) for prolonged periods, ciclofosphamide, metotrexate, leflunomide, cyclosporine, tacrolimus, azathioprine and micofenolate) antiviral prophylaxis with a NA is recommended, starting from the beginning of the immune-suppressive treatment or preferibly 2–4 weeks before. In other conditions patients should be only monitored for HBV DNA reactivation.

If the duration of immunosuppressive therapy is limited, the pharmacologic risk of resistence is diminished; therefore a low cost NA, such as Lamivudine, may be used. In patients who need prolonged immunosuppression the use of more potent NAs at lower risk to induce resistance can be considered).

HBsAg-negative, anti-HBc positive patients. In these subjects HBV DNA should be tested at baseline in order to distinguish real from false OBI. Viremic HBsAg-negative patients should be treated with a NA. Anti-HBc positive subjects with haematological diseases undergoing strongly immunosuppressive treatments such as: fludarabine, dose-dense regimens, autologous or allogenic bone marrow transplant, treatment with monoclonal antibodies (anti – CD-20 and anti CD52) should be treated with a NA (preferably Lamivudine for short term therapies), independently of anti- HBs reactivity.

Anti-HBc positive patients in other clinical settings should not be treated but only monitored for liver enzymes and the emergence of serum HBsAg every 1–3 months. Some experts recommend prophylaxis with a NA also in non-haematological patients if they are treated with anti-CD20.

## Monitoring During Therapy:

Once NAs therapy or prophylaxis has been started, monitoring will essentially be through testing serum HBV DNA and ALT levels every three months, to assess: 1) response to treatment (i.e. reduction of HBV DNA, preferably below the limit of sensitivity of the amplified techniques and ALT normalization) and 2) drug-resistance, which should be suspected in the case of virologic breakthrough while ontreatment, in order to activate an early rescue therapy^3^,^11^. Resistance can be defined clinically by the virologic breakthrough4 but a genotypic testing is reccomended and should be used in order to better define the different mutations and to choose the rescue therapy^3^,^8^.

## Impact on Different Specialist Fields:

Data regarding hepatitis B in immunocompromised patients are very heterogeneous. As a result there is a strong indication to promote studies aimed at defining the natural history of hepatitis B in these patients, to assess – also prospectively - different treatment protocols and to promote close cooperation among different specialists.

## Oncology, Hematology and Hematopoietic Stem Cell Transplantation (HSCT)

### Background:

During chemotherapy hepatitis B can make its appearance in two different phases: 1) during the treatment, in relation to the intense bone marrow suppression, which is associated with a strong viral replication and, sometimes, with the emergence of a fulminant hepatitis in the form of fibrosing cholestasis, 2) after the end of therapy, as during the immuno-reconstitution phase the immune response can bring on a reactivation of hepatitis whose clinical course may be more or less severe depending on the baseline condition of the liver and other possible factors that may contribute to the damage.

In oncology the prevalence of HBsAg-positive patients ranges between 5.3% (in Europe) and 12% (in China). In these patients the frequency of clinical HBVreactivation ranges between 20 and 56%, correlating with the use of steroids, anthracyclines, 5-fluouracil with some virological indicators (presence of HBeAgor of e-minus variants and/or of a detectable HBV DNA prior to therapy). Theclinical significance of relapse has been clearly associated with the pre-chemotherapy liver function, with a mortality of 5–40%.

The reactivation of hepatitis, moreover, influences the continuation of the chemotherapy, inducing its suspension and not infrequently posing problems of differential diagnosis with regard to drug toxicity. Hepatitis B can develop both in active and in inactive carriers and it is generally associated with the reappearance of a significant viremia in the preceding 2–3 weeks.

In hematology the frequency of HBsAg positive patients is higher (12.2% in Greece and 8.8% in a recent study from Italy) and the risk of reactivation appears to be greater than in other settings of oncology, depending on the degree of immunosuppression. In this setting, control of the HBV infection assumes great importance in order to prevent HBV-related complications, but also so as not to modify a highly successful therapeutic schedule. In this field the main prognostic indicators unfavorably associated with hepatitis B reactivation are, besides those already cited, hyper-transaminasemia and the condition of second or third cycle compared to the first^1^,^12^–^14^.

In hematology, a 21–67% (median 50%) risk of reactivation has been described, with an average mortality of 20%. In this setting, the available literature is not clear whether the severity of hepatitis in HBsAg-positive patients is directly due to the liver damage caused by HBV reactivation or by other causes (i.e. VOD, GvHD or MOF) and also the degree of risk in relation to the condition of active or inactive carrier is not clearly determinable.

The risk would appear to be heightened by the use of monoclonal antibodies (antiCD20, antiCD52), with the possibility of hepatitis reactivation (even after a cycle of 1–3 months of prophylaxis with lamivudine) at a variable distance from the last administration of these drugs, particularly in overt carriers, but also in anti-core subjects. An analogous risk exists in the course of allogeneic HSCT, as the immuno-suppressive effect in the conditioning phase is particularly strong and is amplified by the subsequent anti-rejection therapy, so the risk of hepatitis reactivation remains throughout the phase of immuno-reconstitution (in some cases until 1–2 years from transplantation)^1^,^15^–^17^,^45^.

### Experiences in the different virological categories:

1. Active HBsAg-carriers: In the onco-hematological setting lamivudine therapy of chronic hepatitis in active carriers appears to be effective.1
2. Inactive HBsAg-carriers: The start of lamivudine therapy at the time of the clinical relapse (hepatitis) in inactive carriersmaintains a residual mortality of 20%, probably in relation to the baseline conditions and to the delayed treatment.
  However, in retrospective studies lamivudine has been shown to be effective in prophylaxis of hepatitis B (0–9% of hepatitis reactivation compared to 25–85% in untreated patients) and in the only prospective study hepatitis relapse developed in 5% of treated subjects and in 24% of controls. Moreover, in the study the universal use of lamivudine was better than the targeted prophylaxis (activated only at the appearance of HBV DNA with a non-amplified technique, during bimonthly monitoring), both in terms of survival and of hepatitis reactivation (0% vs.53%, *P*=0.002)^1^,^17^,^18^,^49^,^50^.
  Recently many meta-analyses have confirmed the signficant efficacy of lamivudine in preventing hepatitis B in HBsAg positive patients, in reducing deaths and in reducing chemotherapy discontinuation.
  Finally, as lately reported in literature, lamivudine-prophylaxis in HBsAg-positive patients undergoing chemotherapy has been shown to be cost-effectivein terms of HBV reactivation (9.6% LAM+ vs. 43.8% LAM−), liver related deaths (0/500 LAM+ vs 20/500 LAM−), chemotherapy discontinuation and cancer deaths (39/500 LAM+ vs 47/500 LAM−)^46^–^48^.
3. Anti-core patients (HBsAg-negative): In the oncological setting there are few data, at present, for this virological category, which can reach 20–40% in averagely endemic areas and 70–80% in highly endemic areas. However, in the hematological setting, out of a total of 176 patients described in literature, sero-reversion has been reported in 21 subjects (12%) during conventional chemotherapy, whether or not this was associated with HSCT, with percentages of 4–30% during chemotherapy and 14–50% in the course of autologous transplantation.
  After autologous HSCT, hepatitis B developed in anti-HBc patients later (6–52 months, average 19 months) than in overt carriers (average 2–3 months) and none of the patients described died of hepatitis B (in 7 cases during therapy with lamivudine, started at the time of the clinical relapse). After the reactivation nine of the 10 patients remained HBsAg positive and one lost the HBsAg during follow-up. Instead, two deaths out of 39 subjects with seroreversion have been reported in literature after allogeneic HSCTand this appeared to have been significantly linked to the absence of protective antibodies (antiHBs) in the donor and to GVHD^1^.
  Recently the introduction in hematologic treatments of monoclonal anti-lymphocyte B and T antibodies (anti-CD20 and anti-CD52), used alone or together with chemotherapy, has been associatedwith the signaling of some cases of sero-reversion in anti-core subjects, sometimes with a fulminant form and death of the patients, despite therapy with lamivudine^1^. HBV infection has been described to be the most frequently (39%) experienced viral infection in lymphoma patients treated with Rituximab. In a study about 50% of Rituximab-related HBV infections resulted in death, whereas this was the case in only 33% of the patients with other infections.
  An Italian study has lately stressed a very low (1%) overall risk of sero-reversion in a large series of patients treated for lymphoma, but the risk of hepatitis B reactivation was 3.5 fold increased for Rituximab therapy, compared to conventional chemotherapy (*P* < 0.005). Data confirming the increased risk of HBV reactivation in patients undergoing anti B-cell therapy have also emerged in a trial which showed as alemtuzumab containing chemotherapy regimen was associated with a high risk (29%) of reactivation of occult HBV infection and of severe HBV-related hepatitis^51^–^54^.

### Recommendations from the Italian guidelines:

1. In *active carriers* therapy is considered useful to control the liver disease pre- and post-immunosuppressive treatments. In HSCT, in particular, the control of the HBV-related disease permits a more precise diagnosis and treatment of specific liver complications (GVHD and VOD). In these patients, antiviral therapy should be continued lifelong (due to the high risk of relapse after withdrawal) or at least until the disappearance of HBsAg in serum. A strict monitoring of mutants should be activated, in order to prevent hepatitis relapse with rescue therapy.
2. In the *inactive carriers* universal prophylaxis appears to be indicated and should be continued for the entire phase of chemotherapy, until at least 12–18months after the end of the treatment._1_,18 The optimal duration of the prophylaxis is still debated and requires prospective studies. In any case, it is recommended the monitoring of the viremia after suspension, for the prompt diagnosis and return to treatment in the case of reactivation.
3. In anti-HBc positive (HBsAg-negative) patients, two different strategies can be identified: a) in oncology or in patients undergoing mild hematological therapies (judged to be at low immunosuppressive potential, such as the ABVD of the CHOP 21 days scheme), HBsAg monitoring every 1–3 months is advised, with the activation of targeted prophylaxis or therapy in the case of sero-reversion or hepatitis reactivation, respectively. However, the use of HBV DNA monitoring for targeted prophylaxis remains controversial because of the lack of data referred to the timing and duration of the monitoring and to the clinical significance of minimal levels of detectable viremia (i.e. the presence of low levels of serum HBV DNA in OBI carriers after solid organs transplantation has rarely a clinical impacts and is not constantly associated with hepatitis relapse)^19^. b) In subjects who need to be treated with intense immunosuppression (chemotherapy with fludarabine, dose-sense regimes, allogeneic transplant, autologous myeloablative transplant, induction in acute leukemia, use of monoclonal antibodies) universal prophylaxis is proposed.

This approach is strongly indicated in the hematological setting and in patients with signs of a chronic hepatitis (due to a previous history of HBV-related disease and/or to other causes of chronic hepatitis) and/or with a positive serum HBV DNA and/or positive for antiHBe antibodies at the baseline evaluation.

### Effects of different virological conditions in donors (D) and recipients (R) of Allogeneic Hematopoietic Stem Cell Transplantation (HSCT):

1. D HBsAg-/antiHBs+/antiHBc±)->R (HBsAg+):In the case of transplant from an immunized (antiHBs-positive) donor to an overt carrier (HBsAg-positive) recipient two possible scenarios have been described: a) the chance of adoptive transfer of immunity with the possible clearance of HBsAg (especially if recipients are treated with lamivudine), b) an acute and sometimes fulminant hepatitis (in historical series)^1^.
2. D (HBsAg-/antiHBs±/anti-HBc+)->R (HbsAg-/antiHBs±/anti-HBc ±): Only few data are available, indicating that in the case of transplant from an anti-HBc positive donor the risk of sero-reversion in the recipient would appear to be negligible in both anti-HBc positive and negative recipients^21^.
3. D (HBsAg+).>R (HBsAg-): In a few studies, transplant from an HBsAg-positive donor was associated with hepatitis in 44–62% of recipients, with generic hepatic mortality in 33–75% of cases, although the role of HBV in these clinical events was not well defined. In a historical retrospective multicenter study performed in the pre-antiviral phase, the anti-HBV specific immunoglobulins (HBIG) were not protective against the transmission of the infection. In contrast, in a recent study the activation of therapy with lamivudine in donors and of prophylaxis with the same antiviral in recipients significantly reduced the HBV-related hepatitis rate (48 vs. 7%, *P*=0.002) and mortality (24 vs. 0%, *P*=0.01) compared to a historical control group^1^. Furthermore two case reports have confirmed the efficacy of lamivudine-prophylaxis in this clinical setting in preventing HBV related hepatitis^55^,^56^.

### General recommendations in HSCT:

- Vaccination of the recipient prior to transplant, if possible, with accelerated protocols, (recombinant vaccine 40 μg by intramuscular route, time 0-1-2 months or 0-7-21 days), especially if he/she is naïve.
- Vaccination of the donor not immunized prior to transplant, with accelerated protocols (recombinant vaccine 20 μg by intramuscular route time 0-1-2 months or 0-7-21 days) in the case of allogeneic HSCT.
- Treatment of the HBsAg-positive donor with lamivudine pre- HSCT in order to reduce infectivity through the reduction of viremia (preferably below the limit of sensitivity of an amplified assay) and universal prophylaxis of the recipient on the day before the transplant.
- The use of high doses of HBIG (intravenous 10,000 IU) during infusion of hematopoietic stem cells from overt carriers (who have been preventively treated with antivirals) in HBsAg-negative recipients remains controversial.

Because of the actual results of the hepatologic and hematologic therapy there is no reason to deny hematopoietic stem transplantation from an HBV positive donor (any form) if the risk-benefit ratio is in favor of transplantation. Moreover in the case of an HLA identical family HBV positive member there is no point in wasting time and resources in searching for an unrelated donor in the international bone marrow donor bank.

## Dialysis and Solid Organs Transplants (Kidney, Heart and Lung)

### Background:

#### Dialysis:

The incidence of overtcarriers of HBsAg among dialyzed patients is 0–7% in developed countries and 10–20% in developing ones. In these subjects the frequent normality of the transaminase makes clinical judgment difficult, confirming the fundamental role of the virological markers (quantitative HBV DNA) and of the liver biopsy to distinguish between active and inactive carriers (baseline). In this setting data about the condition of OBI carrier among anti-HBc patients are scarce and consider the sole presence of viremia in serum, whose diagnostic sensitivity is low.

In *kidney transplant* the condition of HBsAg carrier can be estimated in 10–20% of cases and is associated with a significantly higher risk of death (OR 2.49, 95% CI), independent of the viremic condition (active or inactive carrier), and the chronic hepatitis presents an accelerated course towards cirrhosis (5.3–12%-year), decompensation and hepatocarcinoma^23^,^24^.

In *heart and lung transplant*, Italian reports have signalled HBsAg positivity in 2.3–3.7% of recipients. In this setting the evolution of the HBV-related disease is accelerated in active carriers and the risk of hepatitis B reactivation post-transplant is over 50% in originally inactive subjects. Finally, the risk of sero-reversion postsurgery (de-novo hepatitis B) in HbsAg-negative/anti-HBc positive recipients seems to be lower than 5%^25^–^27^.

### Clinical experiences in nephrology:

No controlled trials for the treatment of HBV with either interferon or lamivudine in dialyzed patients or in kidney transplants are currently available. Interferon can be used to treat dialyzed patients with chronic hepatitis B, but it is contraindicated in transplanted patients. Short-term administration of lamivudine monotherapy is effective but when the drug is withdrawn, viremia rebounds and hepatitis relapses in most cases. Continuous administration of lamivudine monotherapy for 3 to 4 years is able to obtain long-term suppression of HBV replication and may prevent the development of liver related complications and mortality^28^. Secondary treatment failure is caused by the emergence of YMDD which, in some patients, herald hepatitic flares and progression of the liver disease.

### Recommendations in relation to transplant recipients from the Italian guidelines:

1. *Active carrier:* In candidates for kidney, heart or lung transplant the indication to therapy is confirmed, both in the pre-transplant (with NAs or interferons, when they are tolerated) and in the post-transplant phase (only NAs in view of the high risk of interferon-induced rejection).
2. *Inactive carrier:* Pre-transplant and during dialysis there is no indication for prophylaxis but biochemical and virological monitoring is advised, if the diagnosis has been confirmed by strict adherence to previously defined criteria. Instead, therapy should be used in the reactivated forms (HBV DNA >20,000 IU/ml), especially if associated with significant liver damage (HAI > 4 and/or signs of Þ brotic disease by non-invasive methods). Post-transplant, however, there is an indication to universal prophylaxis, in relation to the available data on mortality in HBV carriers, independently from their virological condition.^23^
3. *Anti-HBc positive recipient*: In these recipients of kidney, heart and lung transplant the presence of subclinical manifestations (low levels of circulating HBV DNA detectable with very sensitive techniques post-transplant) without sero-reversion in over 95% of cases^19^,^23^,^24^,^27^ has been indicated. In this condition only monitoring of the HBsAg is required, with the activation of targeted prophylaxis or therapy onlyin the case of sero-reversionand/or hepatitis, respectively.

### Recommendations in relation to transplant donors:

1. *Anti-HBc positive donors*: In the case of kidney, heart or lung allocation from an HBsAg-negative/anti-HBc ositive/antiHBs-positive or negative donor in a HBsAg-negative recipient, the risk of hepatitis B appears to be less than 5%^27^,^29^. The low risk does not justify preventive prophylaxis, but only HBsAg monitoring (every 3–6 monthsand/or in the case of transaminase increase) and the use of targeted prophylaxis or therapy only in the case of sero-reversion.
2. *HBsAg-positive donors:* In this condition the risk of transmission of the HBV infection is very high in the absence of prophylaxis, especially from HBeAg-positive donors.^30^ Recently some reports have indicated the post-transplant control of hepatitis B in HBsAg-negative/antiHBs-positive recipients of organs from HBsAg-positive donors, while on lamivudine prophylaxis^31^.

## Liver Transplantation

### Background:

The risk of post transplantation hepatitis B is strictly influenced from both recipient and donor virological characteristics:

- *HBsAg-positive recipients:* in the absence of pre-and postoperative prophylaxis the risk of post-transplantation hepatitis B is over 80%. In this condition the use of antivirals before transplant (one single antiviral in the case of wild type virus, combined with a second one that is active on the mutants, in the condition of drug resistance with active replication), associated with HBIG after surgery (combined prophylaxis), is protective in more than 90% of patients^32^,^33^.
- *HBsAg-negative/anti-HBc positive recipients:* in absence of prophylaxis the risk of sero-reversion after transplantation (de-novo hepatitis B) is less than 5% from naïve liver donors and 10–15% from anti-HBc positive donors^19^,^34^.
- *HBsAg-positive donors*: the risk of hepatitis B transmission from a HBsAg-positive donor is high, as the neutralizing effect of HBIG is very low and the reappearance of HDV, in co-infected recipients, is constant. In this particular condition the reactivation of hepatitis would appear to be controlled by the combination of two antivirals in the long term^35^.
- *HBsAg-negative/anti-HBc positive donors:* in this category the overall risk of HBV transmission and hepatitis is high (33–78%), in the absence of prophylaxis, ranging from 70% in naïve to 10–15% in anti-core recipients. Combined prophylaxis with lamivudine±HBIG controls relapse in nearly all cases, while personalized prophylaxis with only HBIG or only lamivudine has been suggested in low risk recipients (anti-core positive)^34^. Comparative studies are not available in this setting.

### Recommendations in relation to recipients:

In all HBsAg-positive carriers there is an indication to universal prophylaxis post-surgery according to their original virological condition:

- *in active carriers*, therapybefore surgery is indicated (with one or two antivirals in cases of YMDD mutants), with the aim of achieving the reduction of HBV DNA below the limit of sensitive HBV assays or at least below < 20,000 IU/ml, in association with combined prophylaxis (HBIG and one or two antivirals, as previously reported) in the post-operative period;
- *in inactive carriers*, the role of therapy before surgery remains controversial because of the high (> 80%) protective effect of post-transplantation combined prophylaxis. In these subjects a preventive reduction of HBV DNA before surgery might not be necessary, with regard to the minimal residual risk, but it could be desirable in order to save HBIG in the long term after liver transplantation. Likewise, insubjects with spontaneous undetectable viremia (PCR-negative) or with levels around the limit of detectability (< 2,000 IU/ml), especially if co-infected with HDV, the protective power of just HBIG seems to be very high. Although also in this conditionthe use of the combined prophylaxis after liver transplantation permits a considerable saving of HBIG in the long term.
- in HBsAg-negative/anti-HBc positive recipients, in analogy with what has been described in the other transplants, albeit in the presence of serum and intra-hepatic evidence of re-infection by HBV in the post-transplant period, the risk of sero-reversion is practically nil^36^–^37^ and so there is no indication for any prophylaxis, but only the monitoring of the HBsAg.

### Recommendations in relation to donors:

The use of organs from HBsAg-positive donors should be considered only in conditions of emergency, avoiding their use in HDV recipients. In this setting the use of universal prophylaxis with two antivirals post-transplant could permit the control of clinical hepatitis B recurrence in the long term. Instead the use of livers from HBsAg-negative/anti-HBc positive donors is justified by the shortage of organs but requires the adherence to specific rules in the Donor/Recipient match (preferential allocation of anti-HBc positive grafts to HBsAg positive or negative/anti-HBc positive recipients) and the activation of universal prophylaxis with lamivudine±HBIG.

## Rheumatology

### Background:

Reports regarding the reactivation of HBV in the rheumatology setting are episodic, during the course of hydroxychlorochine, azathioprine, methotrexate and anti-Tumor Necrosis factor (TNF). The few data available all refer to active and inactive HBsAg carriers. However, reports on anti-CD20 derive from hematological experience, and like in hematology the risk of HBV reactivation in the rheumatology setting would appear to be linked both to the phase of immunosuppression and to that of immuno-reconstitution.

In the meantime no reactivations have been reported in the few HbsAg-positive rheumatology patients undergoing universal prophylaxis with lamivudine during immunosuppressive therapy.^38^–^41^

In the absence of data two risk categories have been identifiedwith regard to the type and to the degree of immunosuppression: a) *high risk* of HBV reactivationin patients undergoing the following therapy: anti-TNF antibodies, medium to high dosage steroids (>7.5 mg/die) for prolonged periods, immunosuppressors such as cyclophosphamide, methotrexate, leflunomide, cyclosporine, tacrolimus, azathioprine and mycophenolate. Although cases of viral reactivation have not yet been described in rheumatology patients undergoing treatment with anti-CD20 antibodies, the data which have emerged in other specialist circles suggest the inclusion in this group of these and other monoclonals; b) l*ow risk* of HBV reactivation in patients treated with steroids at <7.5 mg/die, sulfasalazine and hydroxychlorochine^1^.

### Recommendations from the Italian guidelines:

Among HBsAg-positive patients, therapy is indicated in active carriers and universal prophylaxis with a NA is suggested in inactive carriers who underwent high-risk treatment, especially if they are subjects with manifestations of chronic liver disease due to the previous activity of HBV or other causes. Finally, in inactive HBsAg-carriers treated with low risk therapies and in HbsAg-negative/anti-HBc positive subjects the proposal is a strategy of monitoring, with the activation of therapy or targeted prophylaxis in the case of viral reactivation (HBV DNA > 20,000 IU/ml) or sero-reversion, respectively.

Prophylaxis should be started 2–4 weeks before the immunosuppressive therapy, if possible, and continued for at least 6–12 months afterwards (i.e. after immunosuppressive therapy has been suspended). Hematology literature advises particular caution in suspending prophylaxis, especially in subjects treated with repeated cycles of monoclonal antibodies.

### Peculiar conditions in the rheumatology setting:

Anti-HBV vaccination in rheumatology patients remains controversial and its cost/benefit ratio should be carefully assessed in groups particularly at risk of HBV (for example those living with HBsAg-positive individuals or health workers).

Panarteritis nodosa (PAN) is a rare necrotizing vasculitis that affects small and medium-sized arteries which presents, at least in a portion of cases, a pathogenic correlation with HBV infection. In the treatment of HBV-related PAN, the immunosuppressive therapy (which also poses the question of an uncontrolled activation of the virus) should be associated with an antiviral therapy (in active carriers) or universal prophylaxis (in inactive carriers) to repress viral replication. In this regard single cases and observational studies with small numbers of cases have documented the efficacy of interferon (IFN) and lamivudine.

## HIV

### Background:

Cirrhosis and liver cancer are the second cause of death worldwide in HIV carriers (3–4 million), 9% of whom have HBV infection. Co-infection with HIV increases the rate of chronic HBV infection, reduces the annual rate of seroconversion to antiHBe and to antiHBs and may be linked to the reactivation of the occult infection in HBsAg-negative subjects in the presence of severe immunodepletion.

Moreover co-infection with HIV accelerates progression towards cirrhosis and liver decompensation and reduces survival in decompensated cirrhotics. Therefore mortality due to liver disease in those co-infected with HIV-HBV is higher compared to subjects with just HBV infection^43^–^44^.

### Recommendations from the Italian guidelines:

A. *Patients undergoing Anti-Retroviral viral Therapy* (ART): In active and inactive carrierstherapy and universal prophylaxis with antivirals(utilizing the same NAs effective on HBV used in the treatment of HIV infection) are indicated, respectively. In HbsAg-negative/anti-HBc positive subjects, the condition of occult carrier, characterized by HBV DNA positivity in serum and/or in the liver, has been identified in 35–90% of subjects with HIV co-infection using high sensitivity techniques, and only in 1% of cases with less sensitive techniques. Even in the presence of anecdotal reports of reactivation during immunodepletion and/or of suspension of lamivudine, the risk of sero-reversion appears to be very low (0.23/100 patients/year) and it doesnot therefore justify any prophylaxis but only monitoring^44^.

B. *Patients who do not require ART:* In active carriers therapy with interferons or antivirals is indicated. In these subjects treatment should preferably be administered using drugs which do not have any effect on HIV and which do not, in the future, induce resistance to ART Instead, in inactive carriers and in anti-HBc positive subjects monitoring of HBV DNA or HBsAg, respectively, is recommended, with activation of therapy or targeted prophylaxis in the case of reactivation or sero-reversion.

## Conclusions:

Literature on hepatitis B in immuno-compromised patients is very heterogeneous. It refers mainly to the pre-NAs era and the period prior to the introduction of the modern techniques of determination and quantification of the viremia, which raises many doubts and difficulties about the interpretation of the studies and leaves several aspects still a matter of debate. This encourages a network of communication and studies, in order to better define the natural history, the potential risk of hepatitis B and the results of the various strategies proposed in the management.

Even in the light of such premises today it appears to be justified to propose a rational approach to the problem of hepatitis B in immunocompromised patients, which provides for:a) screening of HBV markers in all subjects starting immunosuppressive therapies and the evaluation of their original liver condition (baseline; b) therapy of active carriers, preferably with third generation NAs; c) prophylaxis, preferentially with a low-cost NA, of inactive carriers and anti-HBc positive patients at risk (onco-hematologic and BMT patients); c) HBV DNA (in inactive overt carriers) or HBsAg (in anti-HBc positive subjects) monitoring of the remaining patients at low risk of reactivation. Finally, in the transplant setting, a precise Donor/Recipient matching should be considered.

## Figures and Tables

**Table 1.** Virological categories

	**Acrive carrier**	**Inactive carrier**	**AntiHBc-positive (anti-core)**
HBsAg	Positive	Positive	Negative
HBeAg	Positive or negative	Negative	Negative
AntiHBs	Negative	Negative	Positive or negative
AntiHBc	Positive	Positive	Positive
ALT^b^	Persistently or intermittently increased	Persistently normal^c^	Persistently normal^c^
HBV DNA serum	≥ 20.000–2.000^a^ IU/ml	< 20.000 IU/ml	Negative (>90%)
HBV DNA tissue	Positive	Positive	Positive
Liver damaged	yes (>90%)	no (>90%)^c^	no^c^

a In anti-HBe positive patients,

b Alanine aminotransferase,

c In the absence of other causes of chronic hepatits and/or of a previous history of chronic hepatits B,

d Necroinflammatory score >4 HAI

**Table 2.** Baseline assessment

**Level 1**	**Level 2**			**Level 3**
	**Patients with altered transaminase**	**Patients HBsAg-positive**	**Patients Anti-core positive**	**Patients HBV DNA-positive and/or with chronic hepatitis**
Transaminase	upper abdomen	HDV	AntiHBe	IgM antiHBc (Imx index)
Colestasis index	US		HBV DNA PCR	Liver biopsy assessment
Hemochrome	Glycemia	HBeAg, AntiHBe		
Total and fractionated bilirubin	Lipidic profile	HBV DNA PCR		
AntiHCV	INR			
HBsAg, AntiHBs titer, AntiHBc	Ferritin			

US ultrasound, INR: International Normal Ratio, PCR: Polymerase Chain Reaction

**Table 3.** Treatment Strategies

**Clinical Condition**	**Original virological condition**			
	**Active carriers**		**Inactive carriers or anti-core positive**	
Infection	yes		Yes	Yes
Hepatitis	yes		No	Yes
**Treatment**	**Therapy**	**Prophilaxis**		**therapy**
		All the population	Only in pateints with infection markers^a^	
		**Universal**	**Targeted**	

a Infectiona markers: evidence of HBV DNA or HBsAg in serum in originally negative patients
